# Transcriptome and proteome profile of jejunum in chickens challenged with *Salmonella* Typhimurium revealed the effects of dietary bilberry anthocyanin on immune function

**DOI:** 10.3389/fmicb.2023.1266977

**Published:** 2023-11-20

**Authors:** Sheng Zhang, Qin Wang, Jinling Ye, Qiuli Fan, Xiajing Lin, Zhongyong Gou, Mahmoud M. Azzam, Yibing Wang, Shouqun Jiang

**Affiliations:** ^1^State Key Laboratory of Livestock and Poultry Breeding, Key Laboratory of Animal Nutrition and Feed Science in South China, Guangdong Provincial Key Laboratory of Animal Breeding and Nutrition, Ministry of Agriculture and Rural Affairs, Institute of Animal Science, Guangdong Academy of Agricultural Sciences, Guangzhou, Guangdong, China; ^2^Department of Animal Production College of Food and Agriculture Sciences, King Saud University, Riyadh, Saudi Arabia

**Keywords:** bilberry anthocyanin, *Salmonella* Typhimurium, immune status, transcriptome, proteome, yellow-feathered chicks

## Abstract

**Introduction:**

The present study investigated the effects of bilberry anthocyanin (BA) on immune function when alleviating *Salmonella* Typhimurium (*S*. Typhimurium) infection in chickens.

**Methods:**

A total of 180 newly hatched yellow-feathered male chicks were assigned to three groups (CON, SI, and SI + BA). Birds in CON and SI were fed a basal diet, and those in SI + BA were supplemented with 100 mg/kg BA for 18 days. Birds in SI and SI + BA received 0.5 ml suspension of *S*. Typhimurium (2 × 10^9^ CFU/ml) by oral gavage at 14 and 16 days of age, and those in CON received equal volumes of sterile PBS.

**Results:**

At day 18, (1) dietary BA alleviated weight loss of chickens caused by *S*. Typhimurium infection (*P* < 0.01). (2) Supplementation with BA reduced the relative weight of the bursa of Fabricius (*P* < 0.01) and jejunal villus height (*P* < 0.05) and increased the number of goblet cells (*P* < 0.01) and the expression of *MUC2* (*P* < 0.05) in jejunal mucosa, compared with birds in SI. (3) Supplementation with BA decreased (*P* < 0.05) the concentration of immunoglobulins and cytokines in plasma (IgA, IL-1β, IL-8, and IFN-β) and jejunal mucosa (IgG, IgM, sIgA, IL-1β, IL-6, IL-8, TNF-α, IFN-β, and IFN-γ) of *S*. Typhimurium-infected chickens. (4) BA regulated a variety of biological processes, especially the defense response to bacteria and humoral immune response, and suppressed cytokine–cytokine receptor interaction and intestinal immune network for IgA production pathways by downregulating 6 immune-related proteins.

**Conclusion:**

In summary, the impaired growth performance and disruption of jejunal morphology caused by *S*. Typhimurium were alleviated by dietary BA by affecting the expression of immune-related genes and proteins, and signaling pathways are related to immune response associated with immune cytokine receptors and production in jejunum.

## 1 Introduction

Intensive animal production is increasingly constrained by bacterial diseases such as *Salmonella, Escherichia coli*, and *Pasteurella*, among which *Salmonella* is particularly prominent in poultry (Foster et al., 2021). In production settings, poultry is infected with *Salmonella* through ingestion of contaminated feed and water as well as by vertical transmission (Karabasanavar et al., 2020). The immune system of chicks is not fully developed, so they are more susceptible to *Salmonella* infection. Infected chicks suffer from weakness, loss of appetite, diarrhea, poor growth, and even death, causing serious economic losses (Mshelbwala et al., 2019; Abudabos et al., 2020). *Salmonella* colonizes and adheres to the intestinal mucosal epithelium after invading the digestive tract, then damages the intestinal barrier function, unbalances the composition of intestinal microbes, and induces intestinal inflammation. During infection, *Salmonella* causes damage to immune organs, resulting in congestion, bleeding, and inflammatory cell infiltration (Chen et al., 2020; Cheng et al., 2020).

Overuse of antibiotics to obviate *Salmonella* invasive infection leads to the emergence of drug-resistant bacteria and is harmful to the environment and health of animals and humans (Manyi-Loh et al., 2018). Nutrition strategies such as dietary supplementation with plant extracts have been introduced into poultry production as alternative substitutes to antibiotics to alleviate *Salmonella* infection (Wu et al., 2018; Purwanti et al., 2019).

Anthocyanins are flavonoid substances obtained from plants such as flowers, fruits, and tubers, which are characterized by excellent antioxidant, anti-inflammatory, and antibacterial activities (Peng et al., 2020; Moreira et al., 2021). Studies showed that anthocyanins alleviated intestinal inflammatory diseases through various mechanisms. Anthocyanins increased the expression of peroxisome proliferator-activated receptor γ (PPARγ) and inhibited the activation of the downstream NF-κB/MAPK signaling pathway, thereby alleviating colonic inflammation on dextran sulfate sodium-induced inflammatory bowel disease (IBD) in mice (Gao et al., 2021). By inhibiting endoplasmic reticulum stress response, anthocyanins inhibited the activation of NOD-like receptor family protein 3 (NLRP3) and the release of IL-1β and IL-18 in lipopolysaccharide (LPS) and adenosine triphosphate treated BV2 microglia cells (Molagoda et al., 2021). In addition, anthocyanins increased the number of epithelial cells and inhibited the infiltration of inflammatory cells in small intestinal mucosa and submucosa, thus alleviating small intestinal epithelial damage in contaminant-induced rats (Chen et al., 2019). Flavonoid substances reduced the adhesion of *E. coli* and *Salmonella* to IPEC-J2 cells, as well as oxidative stress, inflammation, and barrier damage to intestinal epithelial cells (Kovács et al., 2022). These together indicated the potential value of anthocyanins in the alleviation of intestinal inflammation caused by *Salmonella* infection.

Therefore, the objective of this study was to investigate the effects of dietary supplementation with bilberry anthocyanin (BA) on the intestinal morphology and intestinal inflammatory response of chickens challenged with *Salmonella* Typhimurium (*S*. Typhimurium). In addition, jejunal immune function was examined using genomic and proteomic analyses. The study adds to an understanding of the pathogenic mechanism of *S*. Typhimurium and the further development of nutritional strategies against *Salmonella* infection.

## 2 Materials and method

### 2.1 Experimental design and diets

Bilberry anthocyanin (purity >36%) from Tianjin Jianfeng Natural Product R&D Co., Ltd. (Tianjin, China) was used. *S*. Typhimurium (China Center of Industrial Culture Collection 21484, isolated from the pooled heart and liver tissue of 4-week-old chickens), was provided by Professor Weifen Li from Zhejiang University.

**Figure 1:**
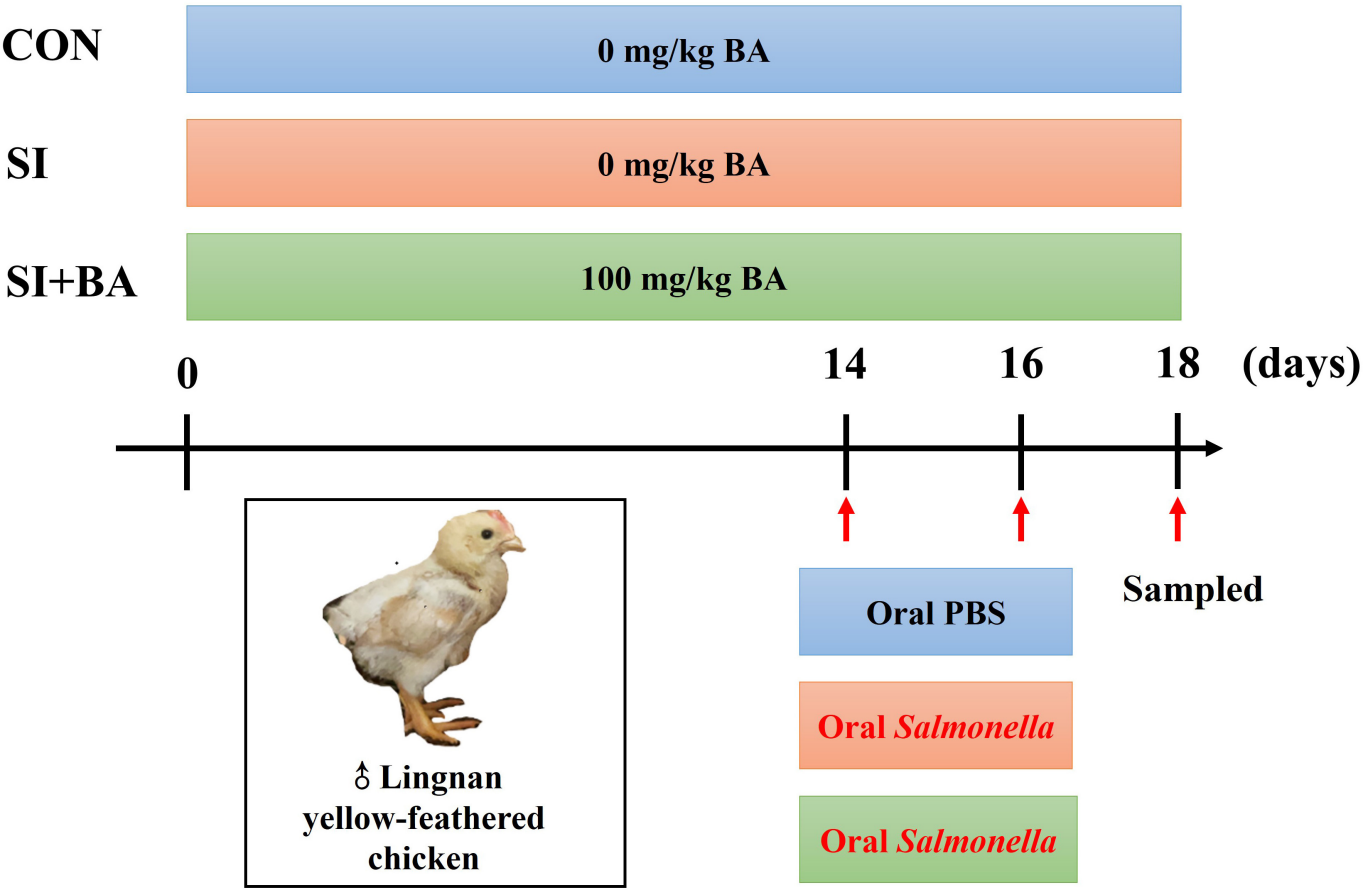
Study design for the whole experiment. CON, control group; SI, *S*. Typhimurium infection group; SI + BA, *S*. Typhimurium infection and dietary BA supplementation group; BA, bilberry anthocyanin.

A total of 180 Lingnan yellow-feathered male chickens (1 day old, 28.00 ± 0.02 g) were randomly assigned to three treatment groups with six cages per treatment (10 birds per cage). As shown in Figure 1, the controls (CON) and birds infected with *S*. Typhimurium (SI) were fed the basal diet for 18 days. The remaining birds (SI + BA) were supplemented with dietary BA at 100 mg/kg. The diets were formulated referring to Chinese Nutrient Requirements of Yellow broilers (Ministry of Agriculture of the People's Republic of China (PRC), 2020), and the composition and nutrient level of the basal corn-soybean diet are shown in Supplementary Table S1. The birds received 0.5 ml sterile PBS (CON) or *S*. Typhimurium suspension (SI and SI + BA) by oral ingestion with a gavage needle gently at 14 and 16 days of age.

The experimental protocol was approved by the Animal Care Committee of the Institute of Animal Science, Guangdong Academy of Agriculture Science, Guangzhou, P. R. China, with the approval number GAASISA-2021-037. Birds were housed and fed in disease-free cages (100 cm × 50 cm × 50 cm) and had unlimited access to feed and water. During days 1–3, the indoor temperature was maintained at ~34°C along with 24-h artificial light; then, the temperature was gradually reduced by 3°C each week to a final temperature of 26°C, and the illumination was reduced by 2 h each day to 16 h. The body weight (BW) of birds was recorded at 14 and 18 days of age.

### 2.2 Preparation of *S*. Typhimurium suspension

*Salmonella* Typhimurium was thawed, streaked on *Salmonella Shigella* agar medium, and placed in a constant temperature incubator at 37°C for 24 h. A single colony was picked and expanded to the exponential phase in Luria–Bertani liquid medium in a shaking incubator (37°C, 180 r/min) for 8 h. The *S*. Typhimurium was washed twice with sterile PBS, quantified by OD_600_, and adjusted to 2 × 10^9^ CFU/ml for oral gavage.

### 2.3 Sample collection and calculation of the relative weight of immune organs

On day 18 of the trial, 12 birds (two close to the average BW from each replicate) from each treatment were electrically stunned (head only) at 150 V for 5 s (DMJ, Ningguang Machinery Co., Ltd., Nanjing, China) and exsanguinated. Blood (5 ml) was collected from the wing vein into anticoagulant (heparin) vacuum tubes and centrifuged at 3,000 × *g* for 10 min to obtain plasma. Jejunal segments (~1 cm long) were quickly fixed in 4% paraformaldehyde. The middle part of the jejunum was opened lengthwise and washed in PBS; then, the mucosa was collected by gentle scraping. Mucosa and intact jejunal wall were frozen in liquid nitrogen and stored at −80°C. The whole wall was used for transcriptome and proteome analyses, and mucosa was used for determining the concentration of cytokines. The spleen, thymus, and bursa of Fabricius were cleaned of connective tissue and weighed. The relative weights of immune organs were expressed based on BW.

### 2.4 Biochemical variables in plasma and jejunal mucosa

Frozen samples of jejunal mucosa were homogenized with ice-cold physiologic saline (1:10, *w*/*v*) and centrifuged at 3,000 × g for 10 min to obtain clarified homogenates. The concentrations of secretory immunoglobulin A (sIgA), IgG, IgM, interleukin (IL)-1β, IL-6, IL-8, tumor necrosis factor-α (TNF-α), interferon (IFN)-β, and IFN-γ in plasma and jejunal mucosa were determined by appropriate ELISA kits (Jiangsu Meimian Industrial Co., Ltd., Jiangsu, China).

### 2.5 Morphology of jejunum

After fixation in 4% paraformaldehyde for 48 h, jejunal segments were trimmed, dehydrated, embedded in paraffin, and sectioned at 5 μm. After mounting and dewaxing, sections were stained with hematoxylin–eosin (H&E) for analysis of morphology and periodic acid–Schiff (PAS) for the observation of goblet cells. The villus height (VH) of five intact intestinal villi in each section and the adjacent crypt depth (CD) were measured, and the number of goblet cells on the villus was counted by scanning browsing software (CaseViewer2.4, 3DHISTECH, Budapest, Hungary) and image analysis software (Image-Pro Plus 6.0). The average of VH and CD and the ratio of villus height to crypt depth (VH/CD) were calculated. In addition, the number of goblet cells on villi was expressed per unit length of the villus.

### 2.6 Quantitative real-time PCR

Total RNA from jejunal samples was extracted using TRIzol^®^ reagent (Invitrogen, Carlsbad, CA). The concentration and purity of the RNA were assessed spectrophotometrically. The RNA integrity number (RIN) was assessed (Agilent 2100 Bioanalyzer, Agilent Technologies, Palo Alto, CA). The RNA was reverse-transcribed with RNAiso Plus and PrimeScriptTMII 1st Strand cDNA Synthesis Kits (6210A, Takara, Tokyo, JP). The real-time PCR was performed using SYBR Premix Ex Taq II (RR820A, Takara) on the CFX96 RT-PCR Detection System (Bio-Rad, Hercules, CA). The primers used are shown in Supplementary Table S2. The abundance of target transcripts was expressed relative to the housekeeping gene (β*-actin*) by the 2^−Δ*ΔCt*^ method and further normalized, relative to data from the CON animals.

### 2.7 Transcriptome analysis

The samples were processed as recommended and then analyzed by Majorbio Bio-pharm Technology Co., Ltd (Shanghai, China) Company, and Majorbio Cloud was used for transcriptome and proteomic analyses (Ren et al., 2022).

The transcriptome library was prepared using 1 μg of total RNA without removing ribosomal RNA. mRNA was isolated from total RNA by A-T base pairing with polyA at the 3′ end of eukaryotic mRNA using magnetic beads with oligo (dT). The extracted mRNA was randomly fragmented by fragmentation buffer, and fragments of ~300 bp were isolated by magnetic bead screening. Then, mRNA was used as the template for reverse transcription into double-stranded DNA. The End Repair Mix was added to patch the end of the double-strand and “A” base to the 3′ end for joining the Y-shaped joint, respectively. After amplification and quantification of PCR, the sequencing was carried out via Illumina NovaSeq6000 sequencer platforms (San Diego, CA). Finally, high-quality sequencing data were selected from the raw sequencing data using fastp (https://github.com/OpenGene/fastp) with default parameters, and the gene expression levels were quantified.

### 2.8 Proteome analysis

Total protein was extracted from the jejunal samples by urea lysis buffer with protease inhibitor, and the concentration of protein was quantified using the Pierce BCA kit (Thermo Scientific Pierce, Rockford, IL). Protein was digested according to the standard procedure, and the resulting peptide mixture was labeled using the 10-plex TMT reagent (90111, Thermo Fisher, Waltham, MA). After desalting with a C18 solid-phase extraction, peptides were used for Nano Liquid Chromatography (EASY-nLC™ 1200, Thermo Scientific)–Mass Spectrometry/Mass Spectrometry (LC-MS/MS) analysis, as previously described (Luo et al., 2019).

### 2.9 Statistical analysis

Shapiro–Wilk test was used to assess the normality of data, and Levene's test was used to assess whether the assumption of homogeneity of variance was fulfilled. The data of growth performance, the relative weight of immune organs, jejunal morphological structure, and concentration of cytokines were analyzed by one-way analysis of variance (ANOVA) followed by Duncan's multiple range tests to compare the individual means in SPSS v20.0 for Windows (SPSS, Chicago, IL). The results are presented as the mean ± SEM. Differences between means were considered to be statistically significant when a *P*-value of < 0.05. For body weight and other variables, values are means of six replicate cages; for transcriptome and proteome analysis, values are the results of three randomly selected samples from each group.

According to the transcripts per million (TPM) reads method, the expression level of each gene was calculated. RSEM (http://deweylab.biostat.wisc.edu/rsem) was used to quantify gene abundance. Differentially expressed genes (DEGs) were identified with |log_2_ fold change (FC)| ≥1 and a *P*-value of ≤ 0.05. The proteomics RAW data files were analyzed using Proteome Discoverer v2.2 (Thermo Scientific). The false discovery rate (FDR) of peptide identification was set as FDR ≤ 0.01. The thresholds of FC (≥1.2 or ≤ 0.83) and *P*-value ≤ 0.05 were used to identify differentially expressed proteins (DEPs). In addition, functional-enrichment analyses including Gene Ontology (GO, http://www.geneontology.org) and Kyoto Encyclopedia of Genes and Genomes (KEGG, http://www.genome.jp/kegg) pathways were performed to identify which DEG and DEP were significantly enriched in GO terms and metabolic pathways at a *P*-value of ≤ 0.05.

## 3 Results

### 3.1 Growth performance and relative weights of immune organs

**Figure 2:**
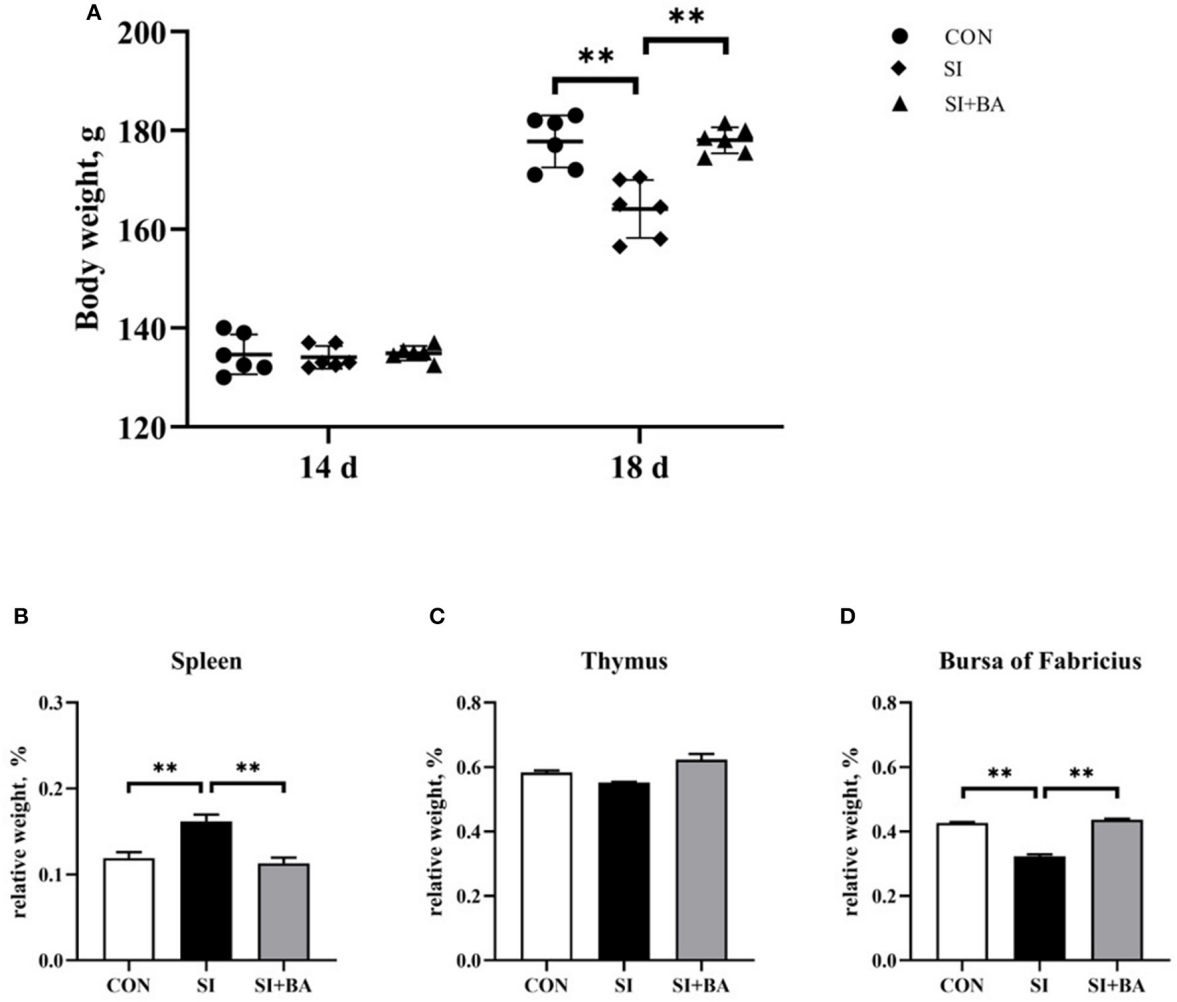
Effect of supplementation with bilberry anthocyanin on the body weight and relative weight of immune organs in chickens challenged with *S*. Typhimurium. **(A)** Body weight. The relative weight of **(B)** spleen, **(C)** thymus, and **(D)** bursa of Fabricius. CON, control group; SI, *S*. Typhimurium infection group; SI + BA, *S*. Typhimurium infection and dietary BA supplementation group. The data are means ± SEM, *n* = 6 (^**^P < 0.01).

As shown in Figure 2A, no significant difference in the total animal BW of chickens at day 14 was observed among the three treatment groups (*P* > 0.05). By contrast, BW at day 18 was significantly reduced in infected birds (SI) compared with the CON (*P* < 0.01), and this suppression of growth was completely relieved in birds given BA (*P* < 0.01).

For the relative weight of immune organs, *S*. Typhimurium infection significantly increased (*P* < 0.01) that of the spleen (Figure 2B) and decreased (*P* < 0.01) that of the bursa of Fabricius (Figure 2D) compared with the CON, and these alterations were significantly offset by BA supplementation (*P* < 0.01). There was no significant (*P* > 0.05) difference in relative thymic weight (Figure 2C).

### 3.2 Morphology of jejunum

**Figure 3:**
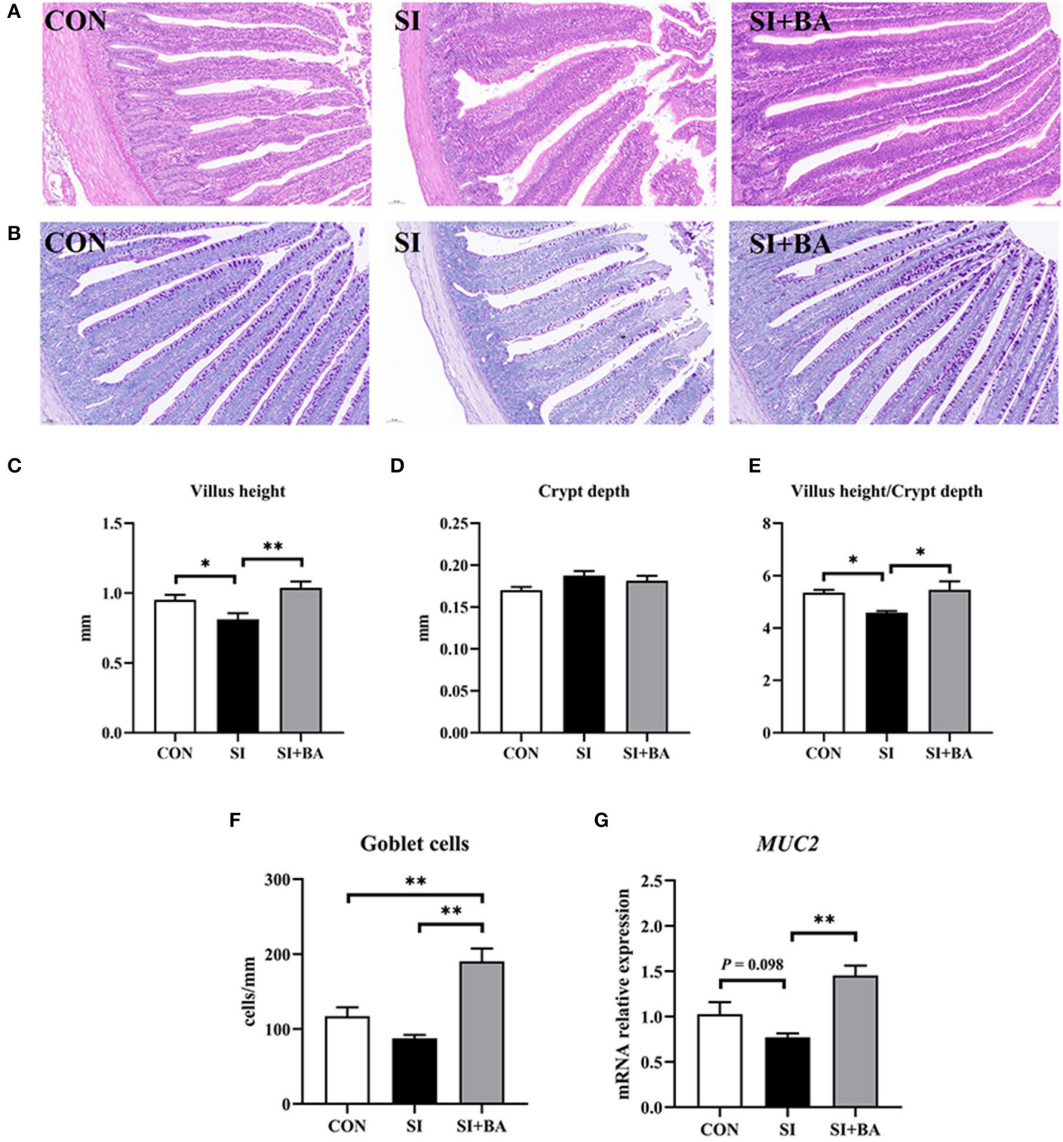
Effect of bilberry anthocyanin on jejunal morphology of chickens challenged with *S*. Typhimurium. Representative images of **(A)** H&E-stained and **(B)** PAS-stained jejunal sections (scale bar at 100 μm). **(C)** Villus height, **(D)** crypt depth, and **(E)** villus height/crypt depth in the jejunum of chickens. **(F)** The number of goblet cells and **(G)** the mRNA expression of *MUC2* in the jejunum of chickens. CON, control group; SI, *S*. Typhimurium infection group; SI + BA, *S*. Typhimurium infection and dietary BA supplementation group; *MUC2, mucin* 2. The data are means ± SEM, *n* = 6 (^*^P < 0.05, ^**^P < 0.01).

Representative images of H&E- and PAS-stained jejunum are shown in Figures 3A, B. Compared with the CON, jejunal VH and VH/CD of birds were decreased (*P* < 0.05) when chickens were challenged with *S*. Typhimurium (Figures 3C, D). Compared with the SI, BA supplementation increased (*P* < 0.01) the VH and VH/CD of birds. No significant (*P* > 0.05) difference in CD existed among the three treatment groups (Figure 3E). In addition, no significant (*P* > 0.05) difference in the number of jejunal goblet cells was noted between birds in the CON and the SI, but that variable was increased (*P* < 0.01) in the SI + BA compared with both SI and CON (Figure 3F). BA supplementation (SI + BA) increased (*P* < 0.01) *MUC2* transcripts compared with those in SI (Figure 3G).

### 3.3 mRNA expression and the concentration of cytokines in plasma and jejunal mucosa

**Figure 4:**
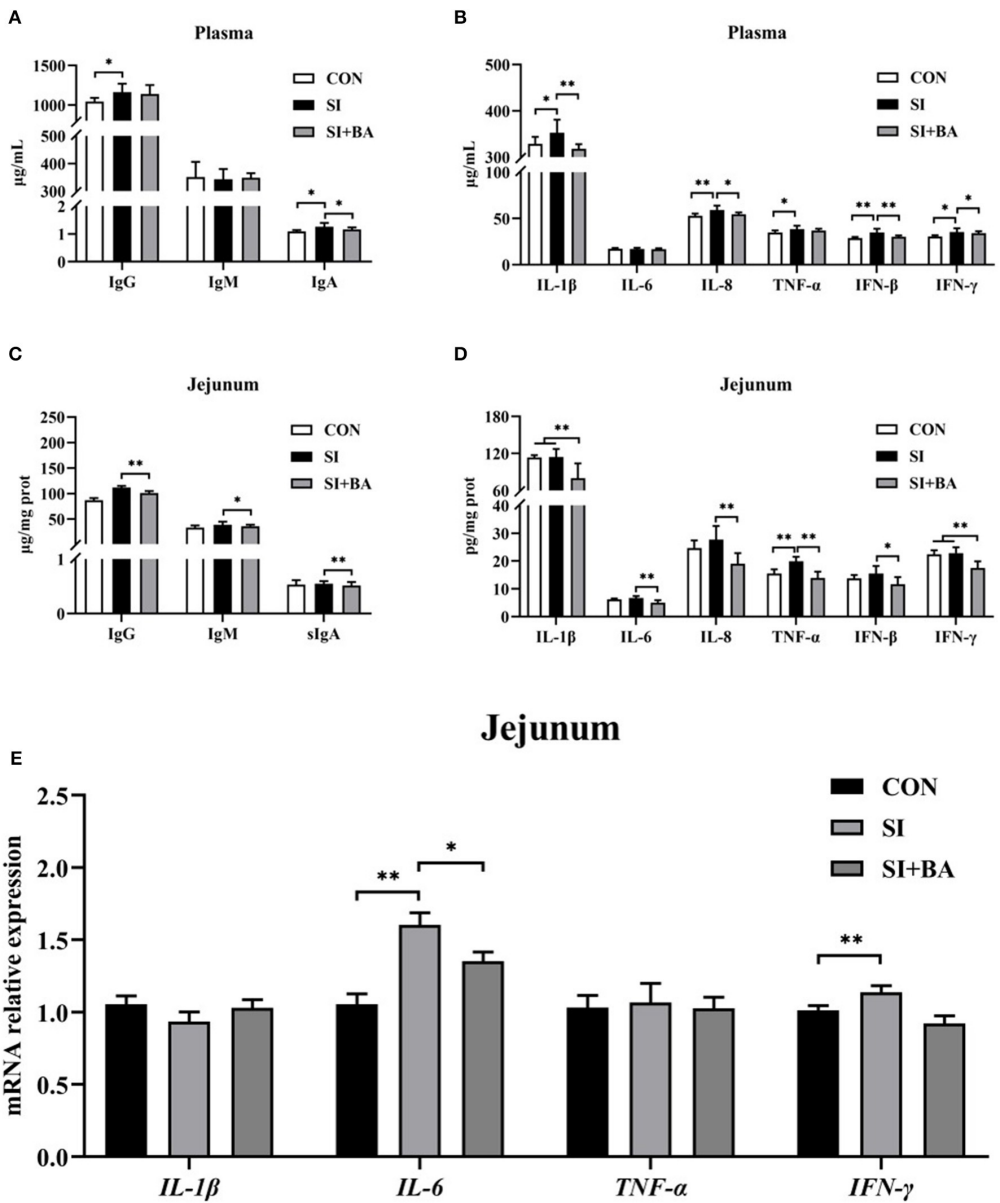
Effect of bilberry anthocyanin on plasma and jejunal mucosal concentrations of cytokines in plasma and cytokine gene expression in jejunal mucosa of chickens challenged with *S*. Typhimurium. **(A)** Plasma immunoglobulins, **(B)** plasma inflammatory cytokines, **(C)** jejunal mucosal immunoglobulins, and **(D)** jejunal mucosal inflammatory cytokines. **(E)** Inflammatory cytokine transcripts in jejunal mucosa: CON, controls; SI, chickens infected with *S*. Typhimurium; SI + BA, chickens supplemented with dietary BA and infected with *S*. Typhimurium; IgG, immunoglobulin G; IgM, immunoglobulin M; IgA, immunoglobulin A; IL-1β, interleukin-1β; IL-6, interleukin-6; IL-8, interleukin-8; TNF-α, tumor necrosis factor-α; IFN-β, interferon-β; IFN-γ, interferon-γ. The data are means ± SEM, *n* = 6 (^*^P < 0.05, ^**^P < 0.01).

As shown in Figures 4A, B, compared with CON, plasma concentrations of IFN-β IL-8 (*P* < 0.01), IgG, IgA, IL-1β, TNF-α, and IFN-γ (*P* < 0.05) increased following *Salmonella* infection (SI). Compared with the SI, BA supplementation (BA + SI) significantly decreased plasma concentrations of IgA (*P* < 0.05), IL-β (*P* < 0.01), IL-8 (*P* < 0.05), IFN-β (*P* < 0.05), and IFN-γ (*P* < 0.01), without affecting (*P* > 0.05) concentrations of IgG, IgM, IL-6, and TNF-α. In addition, *S*. Typhimurium infection increased (*P* < 0.01) jejunal mucosal concentrations of TNF-α, and the concentration of IgG (*P* < 0.01), IgM (*P* < 0.05), sIgA (*P* < 0.01), IL-1β (*P* < 0.01), IL-6 (*P* < 0.01), IL-8 (*P* < 0.01), TNF-α (*P* < 0.01), IFN-β (*P* < 0.05), and IFN-γ (*P* < 0.01) in the jejunal mucosa (Figures 4C, D) of *Salmonella*-infected chickens was significantly decreased by BA supplementation.

Jejunal mucosal transcripts of pro-inflammatory cytokines (*IL*-1β, *IL*-6, *TNF*-α, and *IFN*-γ) are shown in Figure 4E. Compared with controls, *S*. Typhimurium infection stimulated the expression of *IL*-6 (*P* < 0.01), and BA supplementation significantly reduced the expression of *IFN*-γ (*P* < 0.01) and reduced (*P* < 0.05) the expression of *IL*-6 in *Salmonella*-infected chickens.

### 3.4 Identification of immune-relevant mRNA modules

**Figure 5:**
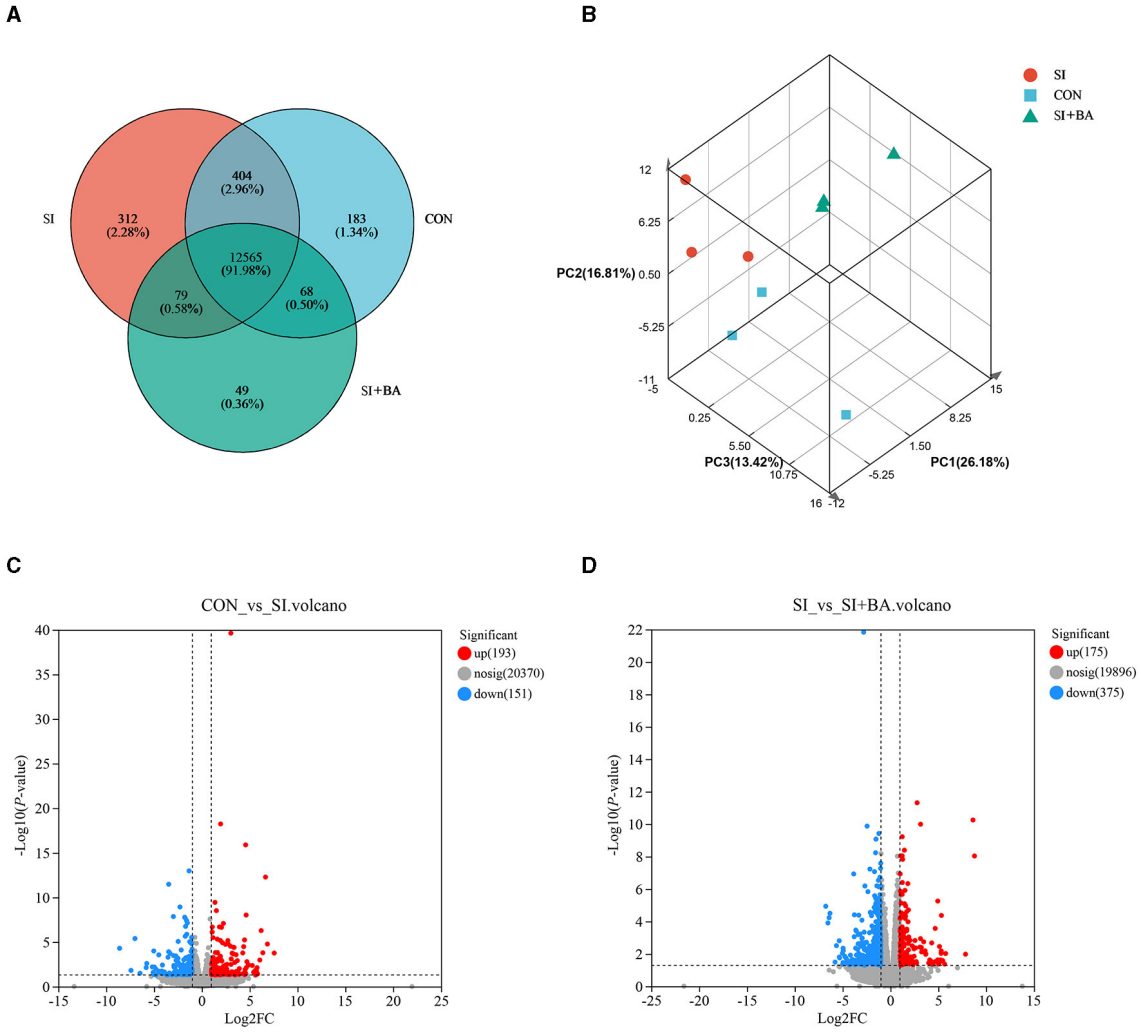
Effect of bilberry anthocyanin on the jejunal global gene expression pattern of chicken challenged with *S*. Typhimurium. **(A)** Venn diagram of the number of genes expressed. **(B)** Principal component analysis (PCA) of each sample on the whole genome. Volcano map of differentially expressed genes about **(C)** CON vs. SI and **(D)** SI vs. SI + BA. CON, controls; SI, chickens infected with *S*. Typhimurium; SI + BA, chickens supplemented with dietary BA and infected with *S*. Typhimurium; PC, principal component; FC, fold change; nosig, no significant change.

As shown in Figure 5A, a total of 13,660 genes were identified and 12,565 genes were shared among the treatments. Principal component analysis (PCA) of the samples was clustered, based on gene expression levels (Figure 5B). Three samples in each treatment group were closely correlated with each other. Furthermore, samples in CON and SI + BA were separated from those in SI, respectively. In addition, the volcano maps (Figures 5C, D) presented a clear visual of the relationship between the FDR and FC for all genes. Of these, 344 genes were differentially regulated (193 up and 151 down) in infected chickens compared with CON, and 550 genes were differentially regulated (175 up and 375 down) in BA-supplemented chickens (SI + BA) compared with those only infected (SI).

**Figure 6:**
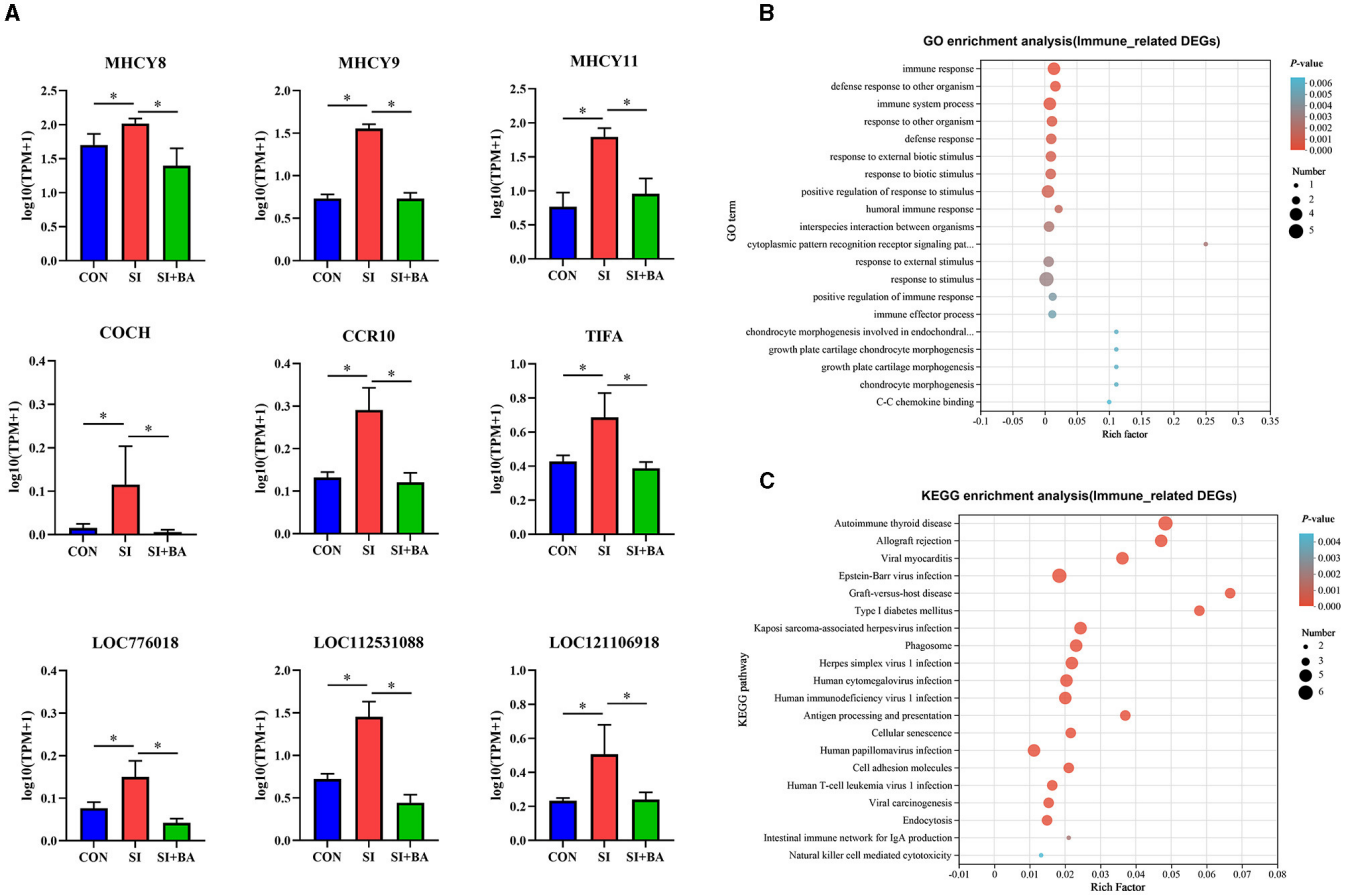
Effect of bilberry anthocyanin on the immune-related genes in the jejunum of chickens challenged with *S*. Typhimurium. **(A)** Differential expression of immune-related genes in jejunum. **(B)** Gene Ontology (GO) and **(C)** Kyoto Encyclopedia of Genes and Genomes (KEGG) enrichment analysis of immune-related DEG. CON, controls; SI, chickens infected with *S*. Typhimurium; SI + BA, chickens supplemented with dietary BA and infected with *S*. Typhimurium. The data in A are means ± SEM, *n* = 3 (^*^P < 0.05).

As shown in Figure 6A, BA supplementation significantly suppressed the upregulation of nine DEG, *viz*. *MHCY*8, *MCHY*9, *MHCY*11, *COCH, CCR*10, *TIFA, LOC*776018, *LOC*112531088, and *LOC*121106918, related to immune regulation triggered by *S*. Typhimurium infection. GO enrichment analysis (Figure 6B) and KEGG pathway enrichment analysis (Figure 6C) were performed for these DEG. GO term at level 2 showed that biological processes such as immune response (GO:0006955), defense response to other organisms (GO:0098542), immune system process (GO:0002376), defense response to a bacterium (GO:0042742), and humoral immune response (GO:0006959) were significantly enriched. The results of the KEGG pathway enrichment analysis indicated that the top five KEGG pathways associated with the nine immune-related DEG in samples included autoimmune thyroid disease (map05320), allograft rejection (map05330), viral myocarditis (map05416), Epstein–Barr virus infection (map00830), and graft-versus-host disease (map05332).

### 3.5 Identification of immune-relevant protein modules

**Figure 7:**
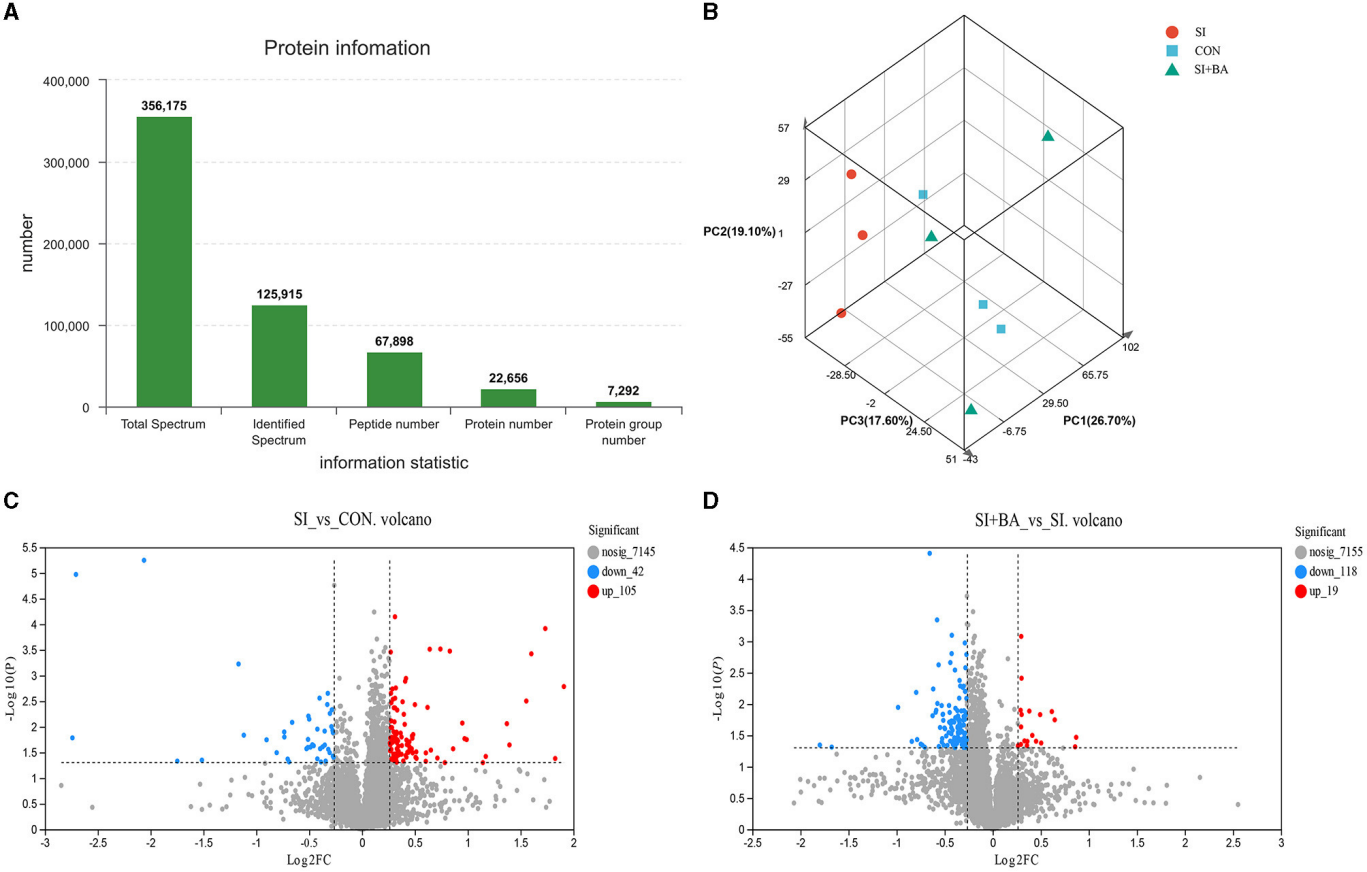
Effect of bilberry anthocyanin on the jejunal global protein expression pattern of chickens challenged with *S*. Typhimurium. **(A)** Histogram of protein information statistics. **(B)** Principal component analysis (PCA) of each sample. Volcano map of differentially expressed proteins about **(C)** SI vs. CON and **(D)** SI + BA vs. SI. CON, controls; SI, chickens infected with *S*. Typhimurium; SI + BA, chickens supplemented with dietary BA and infected with *S*. Typhimurium; PC, principal component; FC, fold change; nosig, no significant change.

In the present study, a total of 7,292 protein groups were identified (Figure 7A) in the jejunum. Based on protein expression levels, PCA (Figure 7B) was performed on samples from the three treatments. The SI showed an obvious separation from the CON and SI + BA. Compared with CON, 147 proteins were differentially expressed (105 were increased and 42 decreased) in *S*. Typhimurium-infected chickens (Figure 7C). In addition, 137 proteins differed in chickens of SI + BA compared with SI with 19 increased and 118 decreased (Figure 7D). The volcano maps presented a clear visual of the relationship between the FDR and FC for all proteins.

**Figure 8:**
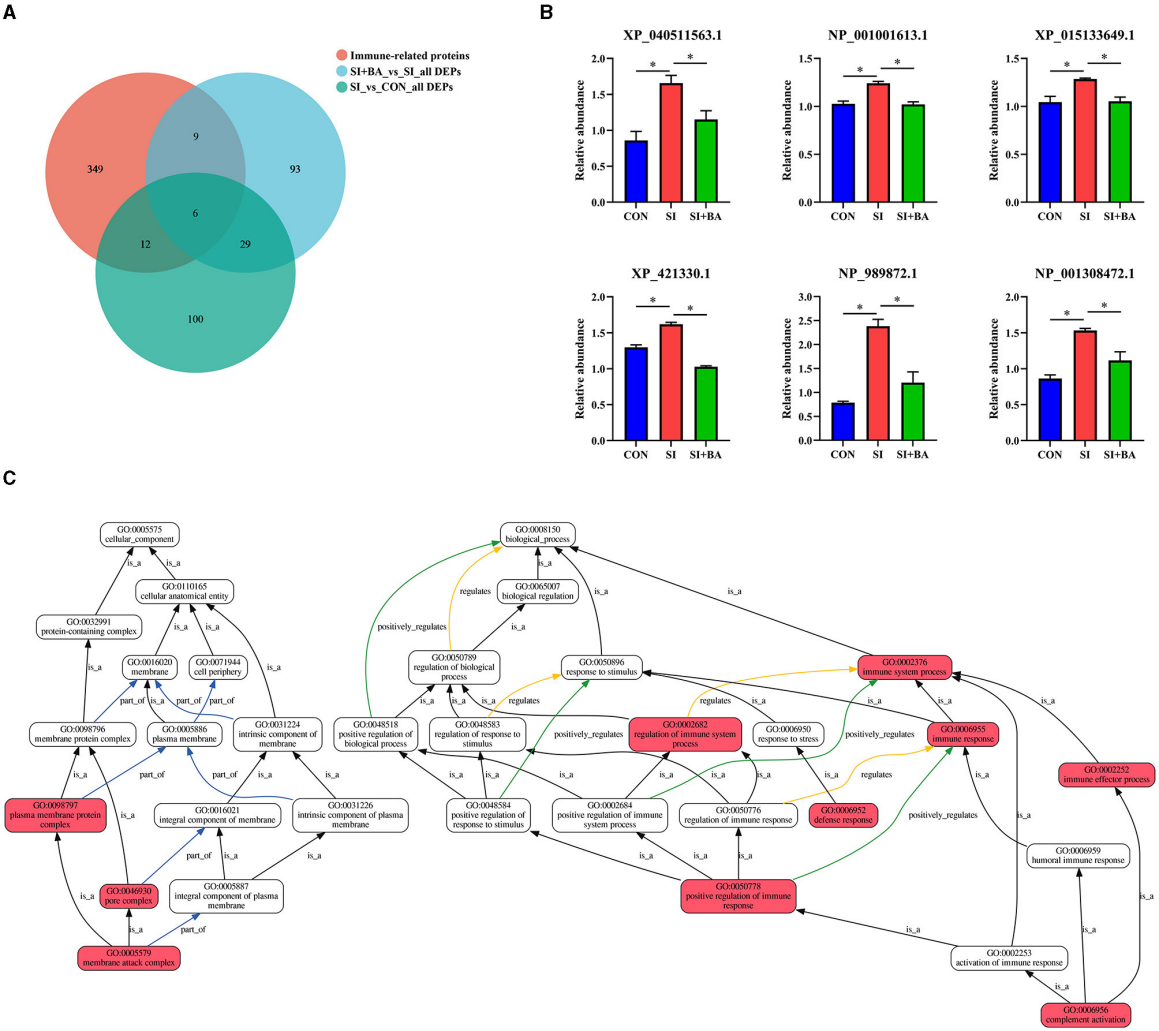
Effect of bilberry anthocyanin on the immune-related proteins in the jejunum of chickens challenged with *S*. Typhimurium. In all parts, CON indicates controls; SI are chickens infected with *S*. Typhimurium; SI + BA are chickens supplemented with BA and infected with *S*. Typhimurium; data in B are means ± SEM, *n* = 3 (^*^P < 0.05). **(A)** Venn diagram of the number of immune-related proteins expressed and those that are differentially expressed. **(B)** Differential expression of immune-related proteins. **(C)** Gene Ontology (GO) enrichment analysis of immune-related DEG. Each box represents a GO term (level 2), and the line indicates the relationship between the two GO terms; the closer it is to red, the more significant it is.

**Figure 9:**
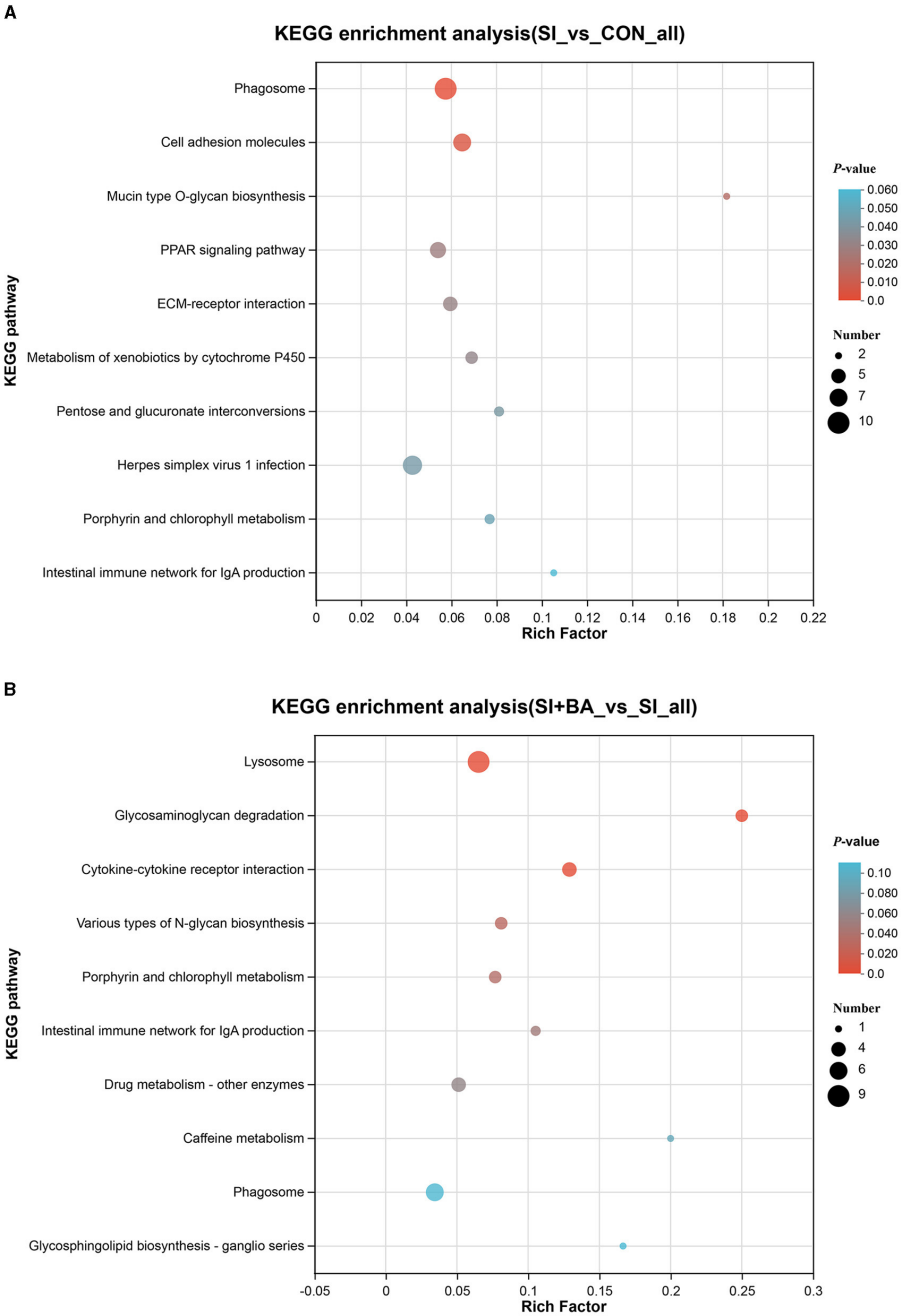
Kyoto Encyclopedia of Genes and Genomes (KEGG) analysis of differentially expressed proteins (DEP) in chickens challenged with *S*. Typhimurium. KEGG enrichment analysis of DEP in **(A)** SI vs. CON and **(B)** SI + BA vs. SI. CON, controls; SI, chickens infected with *S*. Typhimurium; SI + BA, chickens supplemented with BA and infected with *S*. Typhimurium.

As shown in Figure 8A, the number of immune-related DEP (CON and SI, SI + BA and SI) was counted. Six immune-related proteins (Table 1), common to all chickens, were upregulated in response to *S*. Typhimurium infection (Figure 8B); these included orosomucoid 1 (ovoglycoprotein) precursor [fold change (FC) = 3.03], complement component C6 isoform X1 (FC = 1.93), complement component C8 beta chain precursor (FC = 1.78), chromogranin-A (FC = 1.25), tyrosine-protein kinase BTK isoform X1 (FC = 1.23), and HLA class II histocompatibility antigen gamma chain (FC = 1.21), and, moreover, these changes in infected birds were reversed by dietary supplementation with BA. To better understand the function of these 6 DEP, GO enrichment analysis (Figure 8C) showed that immune-related DEP associated with biological processes, such as immune response (GO:0006955), immune system process (GO:0002376), regulation of immune system process (GO:0002682), immune effector process (GO:0002252), defense response (GO:0006952), positive regulation of immune response (GO:0050778), and complement activation (GO:0006956), was upregulated in the SI compared with CON. Cellular components, such as membrane attack complex (GO:0005579), pore complex (GO:0046930), and plasma membrane protein complex (GO:0098797), were upregulated as well. KEGG enrichment analysis information and enriched protein symbol ID were performed in Table 2. The results in KEGG enrichment analysis indicated that phagosome (gga04145), cell adhesion molecules (gga04514), mucin-type O-glycan biosynthesis (gga00512), PPAR signaling pathway (gga03320), and ECM–receptor interaction (gga04512) were significantly (*P* < 0.05) enriched in 147 DEP between SI and CON, and intestinal immune network for IgA production (gga004672) had a tendency (*P* = 0.05) to enrich (Figure 9A). In addition, lysosome (gga04142), glycosaminoglycan degradation (gga00531), cytokine–cytokine receptor interaction (gga04060), various types of N-glycan biosynthesis (gga00513), porphyrin and chlorophyll metabolism (gga00860), and intestinal immune network for IgA production (gga004672) were significantly (*P* < 0.05) enriched in 137 DEP between SI and SI + BA (Figure 9B).

**Table 1:** Immune-related DEP information in the jejunum of chickens challenged with *S*. Typhimurium.

**Accession**	**Description**	**MW, kDa**	**calc.pl**
XP_040511563.1	Complement component C6 isoform X1	104.7	6.92
NP_001308472.1	Complement component C8 beta chain precursor	65.3	7.97
NP_001001613.1	HLA class II histocompatibility antigen gamma chain	31.7	8.31
XP_015133649.1	Tyrosine-protein kinase BTK isoform X1	80.3	7.84
XP_421330.1	Chromogranin-A	53.2	4.49
NP_989872.1	Orosomucoid 1 (ovoglycoprotein) precursor	22.3	5.25

**Table 2:** Kyoto Encyclopedia of Genes and Genomes (KEGG) information in the jejunum of chickens challenged with *S*. Typhimurium.

**KEGG pathway ID**	**KEGG description**	**Associated proteins enriched (symbol ID)**	***P*-value**
**SI vs. CON**			
gga04145	Phagosome	C3, C5, CD36, MHC I, MHC II, NOS3, NOX4, SCARB1, SEC22, SRB1	0.004
gga04514	Cell adhesion molecules	B7H3, B7H4, CES1, PDL1, PTPRF, MHC I, MHC II	0.008
gga00512	Mucin-type O-glycan biosynthesis	GALNT, GCNT2	0.022
gga03320	PPAR signaling pathway	ACBP, CAP, CD36, HMGCS, PCK, SCARB1	0.030
gga04512	ECM–receptor interaction	CD36, LAMA4, LAMB2, LAMC2, SCARB1	0.032
gga00980	Metabolism of xenobiotics by cytochrome P450	AKR7, GST, GSTK1, UGT8	0.034
gga00040	Pentose and glucuronate interconversions	SPR, UGT8, XYLB	0.043
gga05168	Herpes simplex virus 1 infection	C3, C5, CASP7, CD74, HCFC, LZTR1, MHC I, MHC II	0.044
gga00860	Porphyrin and chlorophyll metabolism	HEPH, UGT8, UROS	0.049
gga04672	Intestinal immune network for IgA production	LOC101747454, MHC II	0.060
**SI** + **BA vs. SI**			
gga04142	Lysosome	ACP2, AP3B, AP4B1, CTCS, GALC, GUSB, HEXA/B, IDUA, NPC1	0.001
gga00531	Glycosaminoglycan degradation	GUSB, HEXA/B, IDUA	0.001
gga04060	Cytokine–cytokine receptor interaction	CD30, IL1RAPL, IL1RL2, NGFR	0.002
gga00513	Various types of N-glycan biosynthesis	FUT8, HEXA/B, MGAT4C	0.031
gga00860	Porphyrin and chlorophyll metabolism	GUSB, HEPH, UROS	0.035
gga04672	Intestinal immune network for IgA production	PDCD1LG2, MHCII	0.048
gga00983	Drug metabolism - other enzymes	AOX, GUSB, GMPR, NME	0.057
gga00232	Caffeine metabolism	AOX	0.090

## 4 Discussion

### 4.1 Effect of BA supplementation on growth performance and the relative weight of immune organs in chickens challenged with *S*. Typhimurium

After invading chickens, *Salmonella* transfers from the gut lumen mainly to the spleen and causes intestinal inflammation and systemic infection, resulting in reduced weight gain of chicken (Zhang et al., 2020). *Salmonella* infection also suppresses the development of immune organs, resulting in atrophy and damage of the thymus and bursa of Fabricius (Huang et al., 2016; Ansari et al., 2018). In the present study, *S*. Typhimurium infection significantly reduced the final BW of chickens, similar to previous studies (Wang et al., 2021; Huang et al., 2022). In addition, *S*. Typhimurium infection decreased the relative weight of the bursa of Fabricius and increased that of the spleen. This splenic response was consistent with the report of Wu et al. (2018) and might be related to the transformation and colonization of *Salmonella* into the spleen.

Plants and their extracts such as anthocyanin (Amer et al., 2022), and silymarin (Shanmugam et al., 2022) have been used for the promotion of growth performance and immune status in chickens. Anthocyanins improved growth performance and development of immune organs in mice, including increasing BW and relative weights of thymus and spleen (Yang et al., 2021). Research on DSS-induced colitis in mice showed that dietary anthocyanins restored the BW and feed quantity (Peng et al., 2019). Dietary BA supplementation in *S*. Typhimurium-infected chickens, here, alleviated weight loss and splenomegaly and, similar to Wang et al. (2021), promoted the development of the bursa of Fabricius. These results indicated that BA increased resistance to external pathogens by regulating the state of the immune organs.

### 4.2 Effect of BA supplementation on jejunal morphology and the expression of *mucin* in chickens challenged with *S*. Typhimurium

The intestinal physical barrier and immune functional barrier play an important role in preventing the invasion of pathogens, toxins, and other harmful substances and the diffusion of pro-inflammatory cytokines into the circulatory system (Cornick et al., 2019; Tian et al., 2021). The intestinal tract of chicks lacks an innate immune system; thus, *Salmonella* causes intestinal villus breakage and structural deterioration in chicks more easily and induces intestinal inflammation by damaging intestinal mucosal tolerance after colonization (Zhang et al., 2022). In the present study, *Salmonella* infection decreased VH in the jejunum but BA maintained the integrity and VH of the jejunal villi. Similarly, anthocyanins had an ameliorative effect on intestinal barrier damage from increased intestinal VH with high-fat diet (HFD)-induced colitis in mice (Wang H. et al., 2020) and LPS-induced intestinal inflammation in chickens (Csernus et al., 2020).

*Salmonella* infection reduced the number of goblet cells in the jejunum of chicks by activating the Notch signaling pathway (Xie et al., 2021). Loss of goblet cells regenerates the mucosal layer of intestinal tissue, decreases the secretion of MUC2, and increases intestinal permeability (Ibrahim et al., 2020; He et al., 2021). Mucins, as the first line of defense of intestinal immunity, help to prevent the invasion of pathogens and toxins (Murai et al., 2018; He et al., 2021). In the present study, the number of goblet cells in the jejunum tended to decrease after *S*. Typhimurium infection, while BA supplementation significantly increased the number of goblet cells and upregulated jejunal expression of the *MUC*2 gene. Anthocyanins had previously shown to increase the number of goblet cells in the ileum and colon of HFD-induced mice (Lee et al., 2018; Wang H. et al., 2020) and increased colonic *MUC2* expression in mice (Tian et al., 2019; Wang H. et al., 2020). These are consistent with BA here ameliorating the negative effects of *Salmonella* infection on the number of goblet cells and expression of the *mucin* gene.

### 4.3 Effect of BA supplementation on jejunal inflammatory cytokines in chickens challenged with *S*. Typhimurium

Immunoglobulins and inflammatory cytokines are key molecules in mediating host cell reactions to *Salmonella* infection. IgG, IgM, and IgA are the most common immunoglobulins that are activated during *Salmonella* invasion and released to participate in the elimination of infection (Meijerink et al., 2021). Cytokines (IL-1β, IL-6, IL-8, TNF-α, IFN-β, IFN-γ, etc.), mainly derived from mononuclear phagocytes and other antigen-presenting cells, play important roles in defending against pathogen infection and in promoting infiltration of inflammatory cells in tissues. *S*. Typhimurium infection always results in a strong inflammatory response, accompanied by the secretion of pro-inflammatory cytokines, causing intestinal damage and metabolic abnormalities. *Salmonella* infection in chickens increases the serum concentrations of IgG and IgA (Dar et al., 2019; Song et al., 2020) and IL-1β, IL-6, and TNF-α (Wang G. et al., 2020). *Salmonella* infection increased the expression of *IL*-1β, *IL*-6, and *TNF*-α in the jejunal and ileal mucosa of broilers (Hu et al., 2015; Wu et al., 2018). In the current study, the concentrations of plasma IL-8, TNF-α, IFN-β, IFN-γ, and jejunal TNF-α and IgG were increased in chickens challenged with *S*. Typhimurium. Furthermore, the jejunal expression of *IL*-6 increased, similar to the finding of Song et al. (2020).

Anthocyanins show powerful anti-inflammatory activity and immune regulation function. Wu et al. (2017) found that anthocyanins reduced the concentration of inflammatory cytokines (IFN-γ, L-1β, IL-2, IL-4, IL-6, and IL-10) in the serum of chickens. Furthermore, the anti-inflammatory effect of anthocyanins was also demonstrated by inhibiting the levels of IL-1β, IL-6, TNF-α, and IFN-γ in silica-induced lung tissue injury of mice (Zhao J. et al., 2020), reducing mRNA expression of *IL*-1β, *IL*-6, and *IL*-8 in LPS-induced inflammation in hepatic stellate cell (Lee et al., 2017) and downregulating splenic *IL*-1β mRNA expression in chickens (Csernus et al., 2020). Our previous research found that BA supplementation reduced the levels of plasma IgG and IgM in 63-day-old chickens (Wang et al., 2021). This reduction also occurred here, at day 18, where BA reduced plasma IgA and jejunal IgG, IgM, and sIgA in chicks challenged with *S*. Typhimurium. In addition, BA supplementation decreased the content of pro-inflammatory cytokines, such as IL-1β, IL-6, IL-8, TNF-α, IFN-β, and IFN-γ in jejunum and IL-1β, IL-8, and IFN-β in plasma. These results together indicated that BA mainly alleviated the intestinal stress response to *S*. Typhimurium infection, reduced the intestinal pro-inflammatory cytokines, and improved the intestinal environment, thereby alleviating jejunal inflammatory damage, otherwise caused by *S*. Typhimurium infection.

### 4.4 Effect of BA supplementation on the jejunal transcriptome of chickens challenged with *S*. Typhimurium

In this study, the expression of *MCHY*8, *MCHY*9, *MCHY*11, *TIFA*, and *CCR*10 genes was upregulated by *S*. Typhimurium infection. In addition to classical major histocompatibility complex (MHC), *MHCY* is the second region of polymorphic MHC-like genes associated with bacterial intestinal infection, immune response, and disease incidence (Zhang J. et al., 2021; Goto et al., 2022), the expression of which has a positive correlation with antibody production (Zhang J. B. et al., 2021). In the present study, dietary BA supplementation reduced the expression of *MHCY*, which might be the main reason for suppressing the response to immune challenges and regulating the ability of an individual chicken to respond to *S*. Typhimurium infection.

Activation and oligomerization of TRAF-interacting protein with a forkhead-associated domain (TIFA) were induced by LPS and metabolites produced by Gram-negative bacteria (Gaudet et al., 2017); then, pro-inflammatory cytokines and chemokines were activated via the ALPK1/TIFA signaling axis (Milivojevic et al., 2017; Nasser et al., 2022). In the present study, increased expression of *TIFA* in the jejunum was associated with higher plasma and jejunal contents of TNF-α caused by *S*. Typhimurium infection, similar to the increased phosphorylation and oligomerization of TIFA by TNF-α stimulation (Nakamura et al., 2020). In addition, the increase in plasma IL-8 in the current research was consistent with TIFA-activation promoting IL-8 release (Bauer et al., 2020). As a key regulator of mucosal immune homeostasis, CCR10 is mainly expressed by intestinal IgA^+^ plasmablasts and plasma cells (Zhao L. M. et al., 2020; Davila et al., 2022), directing the migration of IgA^+^ plasma cells to intestinal epithelium (Seong et al., 2017). Increased *CCR* expression was related to the increased IgA, found here in infected chickens. In *Salmonella*-infected mice, the expression of CCR10 was increased in the inflamed gut and neutrophils (Perez-Lopez et al., 2021). Thus, in the present study, the suppression of *TIFA* and *CCR10* expression with BA supplementation of *S*. Typhimurium-challenged chickens was probably associated with the decreased concentration of immunoglobulins and inflammatory cytokines.

From the GO analysis, nine immune-related DEGs were enriched in the defense response and immune response and increased by *Salmonella* infection. Previous examination of immune markers during the progression of *S*. Typhimurium infection showed that the intestinal defense response was related to the control of inflammation (Bescucci et al., 2020). Research in *Salmonella*-infected chicken showed that humoral immunity, as an effective immune response against *Salmonella* infection, provided effective protection to the host (Wang et al., 2022). In this study, dietary BA supplementation decreased the expression of the above nine immune-related DEGs and the corresponding signaling pathways. Anthocyanin regulated the innate immune system and reduced bacterial systemic dissemination in mice challenged with *Klebsiella pneumoniae* (Dong et al., 2021) and was used as a nutraceutical strategy to modulate stress and immune response in vertebrates (Khan et al., 2023).

Taking these results together, dietary BA supplementation reduced the invasion of *S*. Typhimurium by regulating the immune response and alleviating intestinal inflammatory damage by reducing the production of cytokines.

### 4.5 Effect of BA supplementation on the jejunal proteome of chicken challenged with *S*. Typhimurium

Complement proteins are the main components of the innate immune system, enhancing the ability of phagocytic cells to disrupt and clear *Salmonella* pathogens. The activation of complement enables the accumulation of membrane attack complexes on the pathogen cell membrane, ultimately leading to the loss of membrane integrity and the death of the pathogen. The complement components C6 and C8 were involved in the process of lysing the cell membrane of pathogens (Meng et al., 2022). The activity of the HLA class II histocompatibility antigen gamma chain was increased in the current study, which was consistent with CD74 being increased in epithelial cells undergoing an inflammatory response (Balasubramanian, 2022). As a chaperone for the correct folding of MHC class II, HLA class II histocompatibility antigen is involved in regulating the antigen presentation pathway of B cells in the immune response (Noer et al., 2021). In addition, the tyrosine-protein kinase *BTK* gene, which plays a key role in the regulation of B-cell receptor signaling, was identified in the *S*. Typhimurium*-*infected model of mice (Zhang et al., 2018). CgA modulates intestinal barrier permeability and contains unique peptide domains for anti-inflammatory effects (Muntjewerff et al., 2021), and bioactive peptides in the hydrolysates of CgA also have immunomodulatory effects (Muntjewerff et al., 2018). In the present study, two complement proteins (C6 isoform X1 and C8 beta chain precursor), HLA class II histocompatibility antigen gamma chain, and tyrosine-protein kinase BTK isoform X1 and chromogranin-A (CgA) were increased in jejunal mucosa of infected chickens. The secretion of immune-related proteins induced by *S*. Typhimurium infection would cause an adverse response that overactivates the intestinal immune status. These DEPs were associated with the intestinal immune network for IgA production and cytokine–cytokine receptor interaction signaling pathways, consistent with transcriptional findings in the cecal tonsil of chickens challenged with *S*. Typhimurium (Khan and Chousalkar, 2020). The enrichment of the immune-related signaling pathway was involved in the clearance of *S*. Typhimurium in chickens. For example, the cytokine–cytokine receptor interaction signaling pathway was confirmed to be related to the secretion of pro-inflammatory cytokines induced by *S*. Typhimurium infection (Elsharkawy et al., 2022). In addition, the decrease of cytokine responses reduced intestinal inflammation and preserved barrier integrity by *Salmonella*-induced stress of Caco-2 cells (Lépine et al., 2018).

A previous study (Moreira et al., 2021) showed that anthocyanins alleviated LPS-induced stress in mice by suppressing the expression and release of inflammatory cytokines. Consistent with the current study, the expression of the above proteins and the intestinal immune network for the IgA production signaling pathway were downregulated by dietary BA. Secretory IgA is a major immunoglobulin on the mucosal surface against *Salmonella* colonization and invasion (Richards et al., 2021), which protects the intestinal mucosa from infection by binding pathogens and toxins, enhancing the bactericidal activity of monocytes and activating complement production (Adhikari et al., 2019). According to our findings, dietary BA alleviated the immune stimulatory response of *S*. Typhimurium in the jejunum, thus suppressing intestinal inflammatory damage.

## 5 Conclusion

Dietary supplementation with BA alleviated intestinal inflammatory damage in chickens caused by *S*. Typhimurium infection, as evidenced by the improvement of body weight and the decreased content of inflammatory cytokines in plasma and jejunal mucosal. Furthermore, BA addition decreased the jejunal expression of immune-related genes and proteins, associated with the defense response to bacteria and the humoral immune response, pathways of cytokine–cytokine receptor interaction, and the intestinal immune network for IgA production. These ensured the healthy immune status and avoided intestinal damage, otherwise caused by an excessive inflammatory response in chickens challenged with *S*. Typhimurium. The current findings provide a new perspective and insight into poultry production in that BA might be used to alleviate *Salmonella* infection.

## Data availability statement

The data presented in the study are deposited in the NCBI repository, accession number PRJNA1032740.

## Ethics statement

The animal study was approved by Institutional Animal Care and Use Committee, Guangdong Academy of Agricultural Sciences in China (Number: GAASISA-2019-009). The study was conducted in accordance with the local legislation and institutional requirements.

## Author contributions

SZ: Conceptualization, Supervision, Writing—original draft, Writing—review & editing. QW: Investigation, Methodology, Writing—review & editing. JY: Investigation, Methodology, Writing—review & editing. QF: Data curation, Formal analysis, Writing—review & editing. XL: Data curation, Formal analysis, Writing—review & editing. ZG: Resources, Writing—review & editing. MA: Resources, Writing—review & editing. YW: Conceptualization, Methodology, Resources, Supervision, Writing—review & editing. SJ: Data curation, Investigation, Resources, Supervision, Visualization, Writing—review & editing.
