# Supplementary material for: Transcriptome and proteome profile of jejunum in chickens challenged with Salmonella Typhimurium revealed the effects of dietary bilberry anthocyanin on immune function
Source: Front Microbiol. 2023 Nov 20;14:1266977. doi: 10.3389/fmicb.2023.1266977 (PMC10694457; doi:10.3389/fmicb.2023.1266977)
Supplement: Supplementary file 1 [file Table_1.DOCX]

Table S1. Ingredients and nutrient levels of the basal diet at day 1 to 18

|  | Day 1 to 18 |
| --- | --- |
| Ingredient, % |  |
| Corn | 59.20 |
| Corn gluten | 3.00 |
| Soybean meal | 31.00 |
| Soybean oil | 2.30 |
| *DL*-Methionine | 0.10 |
| *L*-Lysine HCL | 0.20 |
| Limestone | 0.85 |
| Monocalcium phosphate | 1.85 |
| Zeolite powder | 0.20 |
| NaCl | 0.30 |
| Premix^1^ | 1.00 |
| Nutrient levels^2^, % or indicated units |  |
| Metabolic energy, MJ/kg | 11.91 |
| Crude protein, | 21.00 |
| Lysine | 1.16 |
| Methionine | 0.43 |
| Methionine + Cystine | 0.78 |
| Calcium | 0.85 |
| Total phosphorus | 0.69 |
| Non-phytic phosphorus | 0.40 |

^1^ Premix provided per kilogram of diet: vitamin A 12000 IU; vitamin D_3_ 600 IU; vitamin E 45 IU; vitamin K_3_ 2.5 mg; vitamin B_1_ 2.40 mg; vitamin B_2_ 5.00 mg; vitamin B_6_ 2.80 mg; vitamin B_12_ 0.02 mg; niacin 42.00 mg; calcium pantothenate 12.00 mg; folic acid 1.00 mg; biotin 0.12 mg; Fe 80.00 mg; Cu 8.00 mg; Mn 80.00 mg; Zn 85.00 mg; I 0.70 mg; Se 0.15 mg.

^2^ The nutrient levels are calculated values (Ministry of Agriculture, RPC 2020).
