# Supplementary material for: Transcriptome and proteome profile of jejunum in chickens challenged with Salmonella Typhimurium revealed the effects of dietary bilberry anthocyanin on immune function
Source: Front Microbiol. 2023 Nov 20;14:1266977. doi: 10.3389/fmicb.2023.1266977 (PMC10694457; doi:10.3389/fmicb.2023.1266977)
Supplement: Supplementary file 2 [file Table_2.DOCX]

Table S2. Primers for real-time PCR

| Gene | GenBank ID | Primer sequences (5’ to 3’) |
| --- | --- | --- |
| *MUC*2 | NM_040673077.1 | F: CATTCAACGAGGAGAGCTGC  R: TTCCTTGCAGCAGGAACAAC |
| *IL-*1*β* | NM_204524.1 | F: GAAGTGCTTCGTGCTGGAGT  R: ACTGGCATCTGCCCAGTTC |
| *IL-*6 | NM_204628.1 | F: TTCGACGAGGAGAAATGC  R: CGAGTCTGGGATGACCACT |
| *IFN-γ* | NM_205149.1 | F: GCCGCACATCAAACACATATCT  R: TGAGACTGGCTCCTTTTCCTT |
| *TNF-α* | MF000729.1 | F: AATTTGCAGGCTGTTTCTGC  R: TATGAAGGTGGTGCAGATGG |
| *β-actin* | NM_205518.1 | F: GAGAAATTGTGCGTGACATCA  R: CCTGAACCTCTCATTGCCA |

*MUC*2, mucin 2; *IL-*1*β*, interleukin-1β; *IL*-6, interleukin-6; *IFN*-*γ*, interferon-γ; *TNF*-*α*, tumor necrosis factor-*α*.
